# Emotion Regulation in Children and Adolescents with Social Anxiety Disorder: Differences in Strategy Use and Repertoire Compared to Specific Phobias and Healthy Controls

**DOI:** 10.1007/s10802-025-01415-w

**Published:** 2026-02-16

**Authors:** Antonia Ikas, Anna-Lina Rauschenbach, Vera Hauffe, Brunna Tuschen‑Caffier, Julian Schmitz

**Affiliations:** 1 Department of Clinical Child and Adolescent Psychology, Institute of Psychology, Leipzig University, Neumarkt 9, Leipzig, 04109 Germany; 2 Department of Clinical Psychology and Psychotherapy, Institute of Psychology, Freiburg University, Engelbergerstr. 41, Freiburg im Breisgau, 79106 Germany

**Keywords:** Emotion regulation, Social anxiety, Children, Adolescents

## Abstract

Both theoretical models and empirical evidence suggest that children and adolescents with social anxiety disorder (SAD) have difficulties with emotion regulation (ER), but little is known about which deficits are disorder-specific or involved across different anxiety disorders for this age group. Furthermore, the available repertoire of ER strategies as an important component of ER flexibility has so far received little attention in research on this age group. Self-reported use of individual ER strategies, the overall repertoire of used ER strategies, and the ratio of adaptive and maladaptive ER strategies were assessed in children and adolescents (aged 10–15 years) with SAD (*n* = 60), clinical controls with specific phobia (SP, *n* = 41), and healthy controls (HCs, *n* = 63) in a cross-sectional study. Children and adolescents with SAD reported using several maladaptive ER strategies (e.g., giving up) more frequently and several adaptive ER strategies (e.g., problem-oriented action) less frequently than both other groups. No group differences in the amount of used ER strategies (repertoire) were identified, but children and adolescents with SAD were found to report a higher ratio of maladaptive and a lower ratio of adaptive ER strategies compared to both other groups. Results suggest that the extent of emotion dysregulation varies with disorder severity, with deficits being more pronounced in children and adolescents with SAD. Potential approaches for SAD treatment, including a shift in repertoire ratios, are discussed.

Social anxiety disorder (SAD) is characterized by an intense and exaggerated fear of negative evaluation by others, frequently leading to the avoidance of social situations (American Psychiatric Association, 2013). With 12-month prevalence rates between 2.3% and 5.2% (Spence et al., 2018; Wittchen et al., 1999), it is one of the most common mental disorders among children and adolescents. However, while there has been increasing research on emotion regulation (ER) processes in the etiology and maintenance of SAD, this has mostly been conducted with adult samples (for a review see Jazaieri et al., 2015). A direct applicability to younger age groups should not be assumed, as ER changes over a person’s lifespan (Gross, 2013) and undergoes major developmental changes during childhood and adolescence (Morris et al., 2007). Although the onset of SAD typically occurs at young ages (Fehm et al., 2008; Kessler et al., 2012), no empirically validated etiological model for childhood SAD is yet available (Hodson et al., 2008). A better understanding of how ER deficits are associated with SAD in children and adolescents, also referred to as youth in the following, could therefore enhance treatment efficacy (Alfano et al., 2002).

## ER in the Context of SAD in Children and Adolescents

In this study, we adopt Gross’s (1998) definition of ER, which refers to “the processes by which individuals influence which emotions they have, when they have them, and how they experience and express these emotions” (p. 275). Emotion dysregulation occurs when individuals, despite their efforts, fail to achieve their emotion-related goals and are not able to adjust their regulatory attempts to achieve those goals (Jazaieri et al., 2013). Difficulties with ER can arise at various stages in the regulatory process (Sheppes et al., 2015), including the selection and implementation of ER strategies.

A large body of research has categorized ER strategies as putatively adaptive or maladaptive based on their associations with affective, social, and other domains as well as different forms of psychopathology (Aldao et al., 2010; Compas et al., 2017; Gross, 2015).

Putatively adaptive ER strategies like *cognitive reappraisal* or *problem-solving* have been shown to reduce the experience of negative affect (e.g., Goldin et al., 2019; Wante et al., 2018), to predict beneficial social outcomes (e.g., English et al., 2012) and to negatively relate to symptoms of anxiety and depression (e.g., Compas et al., 2017; Schäfer et al., 2017). In contrast, strategies considered maladaptive like *suppression* and *rumination* have been found to be associated with a higher persistence of negative emotions (e.g., Campbell-Sills et al., 2006), adverse social outcomes (e.g., English et al., 2012) and higher levels of psychopathology (e.g., Aldao et al., 2010; Compas et al., 2017; Schäfer et al., 2017).

A questionnaire that has adopted this categorization is the FEEL-KJ (Grob & Smolenski, 2005), which has been widely used, also internationally, to assess the self-reported use of ER strategies in children and adolescents (e.g., Cracco et al., 2017; Keil et al., 2017; Lange & Tröster, 2014; Sanchis-Sanchis et al., 2020). Its scale structure, distinguishing adaptive, maladaptive, and other ER strategies, was derived from exploratory factor analyses and confirmed using exploratory and confirmatory factor analyses (Cracco et al., 2015). Different measures of well-being have been shown to correlate positively with the adaptive subscales, such as *problem solving* and *reappraisal*, and negatively with the maladaptive subscales, such as *rumination* and *self-devaluation* (Grob & Smolensky, 2005; Cracco et al., 2015). Studies using the FEEL-KJ found children and adolescents with SAD to use more maladaptive ER strategies like *rumination*, *giving up*, and *withdrawal* more frequently than healthy controls (HCs; Asbrand et al., 2016; Keil et al., 2017; Lange & Tröster, 2014), indicating a rather passive coping style fostering dysfunctional feelings of helplessness. Furthermore, some results also suggest a reduced use of adaptive ER strategies like *acceptance* and *forgetting* in children and adolescents with SAD (Keil et al., 2017; Lange & Tröster, 2014). Preliminary evidence on the causal role of ER deficits in the etiology of SAD was provided by Schneider et al. (2018) in a longitudinal study. Their results suggest that children and adolescents who are lacking certain adaptive ER skills are at an increased risk of developing social anxiety symptoms, although some findings were not specific to social anxiety alone.

Although many studies continue to adopt the concept of putatively adaptive and maladaptive ER strategies, there has also been criticism of the overly rigid categorization of ER strategies (e.g., Aldao, 2013; Bonanno & Burton, 2013). For example, the supposedly maladaptive ER strategy *suppression* has been shown to be effective if the suppression targets the expression as opposed to suppressing the experience of emotion or thoughts of the emotion-eliciting event (Webb et al., 2012). Empirical findings indicate varying efficacy of ER strategies depending on contextual factors (e.g., Carver & Connor-Smith, 2010; Troy et al., 2013), as well as beneficial effects of flexible implementation (Aldao & Nolen-Hoeksema, 2012a; Bonanno et al., 2004; Westphal et al., 2010), leading to the alternative concept of ER flexibility (Aldao et al., 2015; Bonanno & Burton, 2013). One component of ER flexibility is the availability of a broad repertoire of ER strategies (Bonanno & Burton, 2013).

Despite the increasing interest in the overall repertoire of ER strategies, there are only limited findings on its link to psychopathology, specifically anxiety disorders, in children and adolescents. To our knowledge, only two studies have focused on the association between ER strategy repertoire and anxiety in youth. In the first, Lougheed and Hollenstein (2012) found that adolescents who used a limited range of ER strategies reported higher levels of depression, general anxiety, and social anxiety when compared to adolescents with a greater repertoire of ER strategies. In the second study, Quiñones-Camacho and Davis (2019) found children with larger repertoires to have fewer anxiety symptoms in the context of low stress, but more anxiety symptoms in the context of high stress. This implies that in stressful situations it might be more adaptive to routinely use fewer ER strategies.

Most research on ER strategy repertoire and psychosocial functioning, including anxiety symptoms, has focused on adults (e.g., Daniel et al., 2023; De France & Hollenstein, 2017; Grommisch et al., 2020). Some studies indicate a perceived inability to access effective strategies when experiencing negative emotions in socially anxious adults (Rusch et al., 2012). Other studies using ecological momentary assessment (Daniel et al., 2023) and experience sampling (Goodman et al., 2021) suggest that they report using a greater variety of ER strategies per stressful event than HCs. Interestingly, some studies suggest that it is not only the size but a higher ratio of adaptive compared to maladaptive ER strategies that relates to better mental health (e.g., De France & Hollenstein, 2017; Grommisch et al., 2020). Although research on repertoire composition in youth is still scarce, the previously mentioned findings of a heightened maladaptive and reduced adaptive ER strategy use in youth with SAD (Asbrand et al., 2016; Keil et al., 2017; Lange & Tröster, 2014) might suggest a shifted ratio of adaptive and maladaptive ER strategies in their overall repertoire. Research confirming this hypothesized shifted ratio has yet to be conducted but could provide valuable contributions to the understanding of ER deficits in SAD that go beyond singular ER strategies. Considerations about the interaction of ER strategies within the repertoire have furthermore led to two opposing hypotheses: Adaptive ER strategies might either reduce the detrimental effects of maladaptive ER strategies (compensatory hypothesis) or could themselves be impaired in their effect by maladaptive ER strategies (interference hypothesis, Aldao & Nolen-Hoeksema, 2012b). More research is needed to better understand the link between ER strategy repertoire and psychopathology, especially in children and adolescents.

## ER as a Shared Factor in Anxiety Disorders in Childhood and Adolescence

Previous findings indicate that emotion dysregulation is not a feature of SAD but rather a shared phenomenon found in several anxiety disorders (e.g., Cisler et al., 2010). Research further suggests that ER deficits are not specific to anxiety but occur across various forms of psychopathology (e.g., Aldao et al., 2010). This has led to the recommendation to incorporate ER as a sixth domain of the Research Domain Criteria (RDoC) framework (Fernandez et al., 2016), which originally organized research on transdiagnostic factors into five overarching domains (Insel et al., 2010).

However, the question of whether certain ER patterns are disorder-specific to SAD has mostly remained unanswered in previous research. Gaining insights on disorder-specific differences in ER deficits could promote our understanding of the development, maintenance, and treatment of SAD compared to other anxiety disorders in children and adolescents. In the first systematic review on ER in socially anxious children and adolescents, conclusions about disorder specificity of ER deficits were limited by the small number of studies with clinical control groups (Golombek et al., 2020). Keil et al. (2017) compared a clinical group of children with diagnosed SAD to a clinical control group with mixed anxiety disorders (MAD). No significant differences were identified between the two groups regarding the use of individual ER strategies. The overall repertoire and the ratio of adaptive and maladaptive ER strategies were not examined. Since the MAD group included diagnoses of varying severity (specific phobia [SP], separation anxiety disorder, generalized anxiety disorder), the results do not allow for conclusions about a hypothesis proposed in the context of the RDoC framework. According to this hypothesis, the degree of emotion dysregulation varies along a continuum of disorder severity, such that it increases from anxiety diagnoses characterized by circumscribed fears to severe generalized diagnoses with high comorbidities, like SAD (Lang et al., 2016). Studies that test the validity of the hypothesis in the context of anxiety disorders in children and adolescents are still lacking.

## The Present Study

Despite increasing research interest in ER in the context of SAD, particularly the repertoire and ratio of ER strategies, developmental research on children and adolescents with SAD is limited. In addition, previous studies have often not included participants with a clinical diagnosis of SAD (e.g., Lougheed & Hollenstein, 2012; Quiñones-Camacho & Davis, 2019; Schneider et al., 2018). Even fewer allow for a comparison with a homogenous clinical control group, which is essential to test for the disorder-specificity of ER deficits in SAD (Golombek et al., 2020). Moreover, no prior study has examined the overall repertoire of ER strategies in youth with SAD, nor the ratio of adaptive and maladaptive strategies within this repertoire.

In response to this research gap, the present study’s aim was to examine the use of adaptive and maladaptive ER strategies, the overall ER strategy repertoire, and its composition in a sample of children and adolescents with clinical SAD, a clinical control group with SP, and HCs. Specific phobias (SP) are defined by intense, persistent fears of particular objects or situations that go beyond normative developmental fears (American Psychiatric Association, 2013). They served as a clinically relevant comparison group, allowing us to examine whether ER difficulties differ between anxiety disorders with more circumscribed versus more generalized presentations. This comparison, based on evidence from RDoC-informed research (Lang et al., 2016), provided a rationale for expecting, on average, greater disorder severity and thus greater ER deficits in youth with SAD compared with those with SP. We chose an age range of 10 to 15 years to investigate SAD during a critical developmental window, since the average age of onset for SAD is 13 years (Kessler et al., 2005).

First, based on previous findings (Asbrand et al., 2016; Keil et al., 2017; Lange & Tröster, 2014), we hypothesized that youth with SAD and SP use adaptive ER strategies less frequently and maladaptive ER strategies more frequently than HCs. In both cases, drawing on Lang et al.’s findings (2016), we expected the differences to the HCs to be less pronounced in the SP group. Since a small repertoire of ER strategies has been assumed to be a sign of limited flexibility and thus of emotion dysregulation (Bonanno & Burton, 2013), we further hypothesized that youth with SAD would have the smallest and HCs would have the largest repertoires, while those with SP were expected to be located between the other groups. In line with our previous hypotheses on adaptive and maladaptive strategy use, youth with SAD were expected to show the lowest ratio of adaptive and the highest ratio of maladaptive ER strategies. The opposite pattern was expected for HCs. Again, those with SP were expected to lie between the other two groups in terms of both the ratio of adaptive and maladaptive ER strategies.

## Method

### Recruitment and Procedure

Recruitment and data collection took place between 2021 and 2023. The sample consisted of *n* = 164 German children and adolescents (49% female) aged 10 to 15 years, who were recruited in two centers as part of a larger two-center project via local schools as well as the cities’ registers of residents. The inclusion criterion was a primary diagnosis of social anxiety disorder for the SAD group (*n* = 60) and a primary diagnosis of specific phobia for the SP group (*n* = 41), with both diagnosed according to the DSM-5 (American Psychiatric Association, 2013). For the HC group (*n* = 63), any past or present mental disorder was considered an exclusion criterion. Further exclusion criteria that applied to all three groups were a current or past psychotic episode, severe depressive episode, autism spectrum disorder, suicidality, current or past psychotherapeutic treatment, psychotropic drug intake, pervasive developmental or neurological disorders, and strong visual impairments.

After an initial telephone screening, eligible children and adolescents and their parents were then interviewed separately using the “Structured Diagnostic Interview for Mental Disorders in Children” (Schneider et al., 2017), whose reliability and validity has been confirmed (e.g., interrater reliability for lifetime anxiety disorders, children: *κ* = 0.90, parents: *κ* = 0.94; Neuschwander et al., 2013). Trained advanced graduate or doctoral students conducted the diagnostic interviews, which were videotaped and supervised by licensed clinical psychologists. Reports of both interviews and questionnaire data were then integrated to arrive at a diagnosis. A diagnosis was assigned if criteria were fulfilled in the child report or in both child and parent reports. Parental report alone warranted a diagnosis only if the child also reported subclinical symptoms. Suggested diagnoses by the interviewers were confirmed by a licensed clinical psychologist or discussed in the few cases of ambiguity to find a consensus. All participants and their parents were informed about the three-session project procedure (session 1: diagnostic interview; session 2: eyetracking [Hauffe et al., 2025]; session 3: two EEG experiments [Rauschenbach et al., 2024; Rauschenbach et al., 2025]). Before the diagnostic process began, parents provided written informed consent and children and adolescents provided assent. Children and adolescents received age-appropriate vouchers worth €70, and parents were paid €30. Approval for the project was granted by the ethics committees of both Leipzig University (approval number: 037/19-ek) and the University of Freiburg (approval number: 125/19).

### Instruments and Materials

#### ER Strategies

The German questionnaire “Fragebogen zur Erhebung der Emotionsregulation bei Kindern und Jugendlichen” (FEEL-KJ; Grob & Smolenski, 2005) is a self-report measure that assesses children’s and adolescents’ habitual use of ER strategies in response to anxiety, sadness, and anger. In this study, only the anxiety scale was used. The FEEL-KJ measures 15 ER strategies, each assessed by two items. Seven ER strategies are classified as adaptive (*problem-oriented action*, *cheering up*, *distraction*, *acceptance*, *problem solving*, *forgetting*, *reappraisal*), five strategies are categorized as maladaptive (*withdrawal*, *self-devaluation*, *giving up*, *rumination*, *aggressive action*), and three strategies are classified as neither adaptive nor maladaptive (*social support*, *expression*, *emotional control*). Children and adolescents were asked to rate the frequency of their habitual use of a strategy on a five-point Likert scale (1 = almost never, 2 = rarely, 3 = occasionally, 4 = often, 5 = almost always).

The FEEL-KJ has acceptable psychometric properties (Grob & Smolenski, 2005), with the internal consistencies of the individual scales ranging from satisfactory (α = 0.69) to excellent (α = 0.91) and a good retest-reliability after 6 weeks (0.62 ≤ rtt ≤ 0.81). The reliability and construct validity of the FEEL-KJ have been confirmed in international samples (Cracco et al., 2015; Pazos Siri et al., 2023). In the present study, internal consistency of the anxiety scale was good for adaptive strategies (α = 0.87) and acceptable for maladaptive strategies (α = 0.75). Internal consistencies of the individual scales ranged from α = 0.36 to α = 0.89 (Table 1).

**Table 1:** Internal consistencies of the subscales of the FEEL-KJ in the present sample

	Cronbach’s α
Primary scales	
Adaptive	
Problem-oriented action	0.62
Distraction	0.87
Cheering up	0.80
Acceptance	0.48
Forgetting	0.50
Problem solving	0.72
Reappraisal	0.56
Maladaptive	
Giving up	0.43
Aggressive action	0.44
Withdrawal	0.73
Self-devaluation	0.70
Rumination	0.36
Other strategies	
Social support	0.89
Expression	0.81
Emotional control	0.67

#### ER Strategy Repertoire

Since the FEEL-KJ does not directly provide a measure of the ER strategy repertoire, the repertoire needed to be operationalized based on the FEEL-KJ data. This was achieved in two steps. First, a dichotomous categorial variable was created for every single ER strategy of the FEEL-KJ. Dichotomizing frequency ratings of ER strategy use to create a repertoire variable has been applied in previous studies (Aldao & Nolen-Hoeksema, 2013; Cummings et al., 2023). However, since we had to combine the ratings of two items per strategy, our dichotomization procedure was idiosyncratically developed and needs further validation. If a participant rated the frequency of a strategy as 3 (occasionally) or higher on both items of a strategy, the strategy was considered to be used and was assigned a value of 1 on the corresponding dichotomous variable. If at least one of the two items of a strategy was rated lower than 3, the strategy was considered not to be used, resulting in a value of 0. This resulted in 15 dichotomous variables, one for each of the ER strategies assessed by the FEEL-KJ. In a second step, a sum score across these 15 variables was created, representing the total ER strategy repertoire of a participant and resulting in possible integer values between 0 and 15.

#### Depression

The German version of the self-report questionnaire “Children’s Depression Inventory” (CDI; Stiensmeier-Pelster et al., 2014) assesses the severity of depressive symptoms in children and adolescents. It consists of 29 items covering the diagnostic criteria of the DSM-5 (American Psychiatric Association, 2013) as well as typical accompanying symptoms. For each of these items, participants had to choose one out of three pre-formulated answers with scores ranging from 0 to 2. The sum score across all items of the CDI represents the severity of depressive symptoms. According to the manual (Stiensmeier-Pelster et al., 2014), internal consistency has been shown to be excellent in a clinical sample (α = 0.93) and good in an unselected school sample (α = 0.87). Internal consistency was good in the present sample (α = 0.90).

#### Additional Psychometric Measures

To further describe the sample, we assessed social anxiety symptoms using the German version of the “Social Anxiety Scale for Children-Revised” (SASC-R-D; Melfsen & Florian, 1997). Consisting of 18 items, which are rated on a five-point Likert scale, the SASC-R-D has been shown to have satisfactory retest reliability (after 2 weeks: 0.74 ≤ rtt ≤ 0.84) and internal consistency (α = 0.63–0.83; Melfsen & Florian, 1997). Phobic fears were assessed using the German version of the “Fear Survey Schedule for Children – Revised” (FSSC-R; Döpfner et al., 2006). For each of the 96 items grouped into 7 subscales (e.g., fear of animals), children and adolescents rated their level of fear toward an object or a situation. The FSSC-R has good reliability and validity, with internal consistencies ranging from α = 0.70 to 0.93 for the subscales and the total scale (Döpfner et al., 2006).

Furthermore, parents were required to fill out the German version of the “Child Behavior Checklist” (CBCL; Döpfner et al., 2014), which measures emotional and behavioral problems in children and adolescents. The CBCL has been shown to have good to excellent test-retest reliability and high internal consistency (Achenbach & Rescorla, 2001).

### Statistical Analyses

To assess group differences in individual adaptive and maladaptive ER strategies, a multivariate analysis of variance (MANOVA) was conducted with Group (SAD, SP, HC) as the independent variable and 12 ER strategies of the FEEL-KJ as dependent variables, including only the strategies categorized as adaptive or maladaptive. With regard to previous evidence on age and gender effects (e.g., Hampel & Petermann, 2005; Zimmermann & Iwanski, 2014), a three-way MANOVA including Gender and Age group (aged 10–12 years = young, aged 13–15 years = old) was evaluated a priori, but did not reach significance (*ps* > 0.055).

Second, a Poisson regression analysis was conducted for the ER strategy repertoire. To control for potential confounding influences, Age and Gender of children and adolescents as well as the centered CDI sum score as a measure of depressive symptoms (Lougheed & Hollenstein, 2012) were included in the first regression step. In the next step, the Group variable was added as a predictor.

Finally, for each of the participants who had at least one strategy in their ER strategy repertoire, percentages of adaptive, maladaptive, and other strategies in the repertoire were calculated. This led to the exclusion of one HC child with a repertoire of zero strategies. Two one-way analyses of variance (ANOVAs) with Group (SAD, SP, HC) as the predictor were conducted. In the first ANOVA, the dependent variable was the percentage of adaptive strategies in the repertoire sum score, while in the second ANOVA, the dependent variable was the percentage of maladaptive strategies in the repertoire sum score.

To further explore the observed group differences, we conducted exploratory analyses to investigate associations between symptom severity, assessed by sum scores of psychometric measures (SASC-R-D, FSSC-R, CDI, CBCL), and overall adaptive and maladaptive ER strategy use, as indexed by sum scores of adaptive and maladaptive subscales of the FEEL-KJ.

All statistical analyses and figure creation were carried out with R (Version 1.4.1106). For all statistical tests, a value of *p* <.05 was considered statistically significant. Partial eta squared ($\:{{\upeta\:}}_{\mathrm{p}}^{2}$) served as a measure of effect size for the MANOVA and eta squared ($\:{{\upeta\:}}^{2}$) for the ANOVA. These effect sizes were interpreted according to Cohen’s guidelines of 0.01, 0.06, and 0.14 for small, medium, and large effects, respectively (Cohen, 1988). Pseudo $\:{R}^{2}$ was calculated as a measure of effect size for the Poisson regression (Coxe et al., 2009). For pairwise comparisons between the groups, post hoc Tukey tests were performed.

## Results

### Sample Characteristics

Sample characteristics are presented in Table 2. As expected, the SAD group showed a significantly higher level of social anxiety symptoms compared to both control groups. Youth with SP significantly differed from both groups in terms of phobic fears, with their scores being higher than those of the HC group and lower than those of the SAD group.

**Table 2:** Sample characteristics

	SAD (*n* = 60)	SP (*n* = 41)	HC (*n* = 63)	Statistics		
	*n* (%)	*n* (%)	*n* (%)	$\:{{\upchi\:}}^{2}$(df = 2)		
Gender (female)	31 (51.67)	20 (48.78)	30 (47.62)	0.21; n.s.		
Comorbidities						
Specific Phobia	30 (50.00)	–	–			
GAD	8 (13.33)	–	–			
Separation Anxiety	2 (3.33)	–	–			
OCD	1 (1.67)	–	–			
ADHD & Tic Disorders	5 (8.33)	1 (2.44)	–			
Depressive Disorders	4 (6.67)	–	–			
Sleep Disorders	1 (1.67)	–	–			
Elimination Disorders	1 (1.67)	2 (4.88)	–			
ODD	2 (3.33)	–	–			
Selective Mutism	1 (1.67)	–	–			
	*M* (*SD*)	*M* (*SD*)	*M* (*SD*)	*F*(2, 161)	Post hoc Tukey	
Age	12.57 (1.73)	12.22 (1.54)	12.49 (1.67)	0.56; n.s.		
FSSC-R	63.97 (23.24)	46.46 (25.44)	25.59 (17.50)	47.64***	SAD > SP > HC***	
SASC-R-D	52.73 (11.16)	36.27 (9.06)	32.81 (7.34)	77.21***	SAD > HC***	
					SAD > SP***	
CDI	16.82 (7.94)	9.02 (5.44)	6.21 (4.99)	45.3***	SAD > HC***	
					SAD > SP***	
CBCL total	34.48 (18.48)	18.98 (12.90)	8.40 (6.17)	58.13***	SAD > SP > HC***	
Internalizing	16.52 (8.90)	7.66 (5.91)	2.40 (2.41)	77.71***	SAD > SP > HC***	
Externalizing	6.63 (5.24)	4.17 (3.19)	2.62 (2.96)	15.68***	SAD > HC***	
					SAD > SP**	

*Notes.* *SAD* social anxiety disorder, *SP* specific phobia, *HC* healthy control, *GAD* generalized anxiety disorder, *OCD* obsessive compulsory disorder, *ADHD* attention-deficit/hyperactivity disorder, *ODD* oppositional defiant disorder, *FSSC-R* Fear Survey Schedule for Children, *SASC-R-D* Social Anxiety Scale for Children-Revised, *CDI* Children’s Depression Inventory, *CBCL* Child Behavior Checklist

** *p* <.01

*** *p* <.001

### ER Strategies

The MANOVA revealed a significant main effect of Group, Pillai’s Trace = 0.57, *F*(24, 302) = 4.96, *p* <.001, $\:{{\upeta\:}}_{\mathrm{p}}^{2}$ = 0.28, indicating a large effect. The post hoc Tukey test revealed less frequent use for the adaptive ER strategies *problem-oriented action*, *distraction*, *cheering up*, *acceptance*, and *problem solving* and more frequent use of the maladaptive ER strategies *giving up*, *withdrawal*, and *rumination* in the SAD group when compared to the HC group (see Table 3). Compared to the SP group, youth with SAD reported using the adaptive strategies *problem-oriented action*, *distraction*, and *cheering up* significantly less frequently, while using the maladaptive strategies *giving up*, *withdrawal*, and *self-devaluation* more frequently. Whereas for the adaptive strategies, no significant differences were found between the SP and the HC groups, youth with SP reported using the maladaptive strategies *giving up* and *rumination* more frequently than the HC group.

**Table 3:** Group differences in ER strategies

	SAD (*n* = 60)	SP (*n* = 41)	HC (*n* = 63)	Statistics		
	M (SD)	M (SD)	M (SD)	F(2, 161)	Post hoc Tukey	d [95% CI]
Primary scales						
Adaptive						
Problem-oriented action	2.78 (0.95)	3.33 (0.94)	3.69 (0.99)	13.71***	SAD < HC***	0.93 [0.54, 1.38]
					SAD < SP*	0.58 [0.21, 1.03]
Distraction	2.88 (1.14)	3.65 (0.94)	3.86 (1.07)	13.99***	SAD < HC***	0.89 [0.50, 1.31]
					SAD < SP**	0.72 [0.30, 1.22]
Cheering up	2.68 (1.05)	3.29 (1.19)	3.57 (1.03)	10.70***	SAD < HC***	0.85 [0.47, 1.29]
					SAD < SP*	0.55 [0.17, 1.00]
Acceptance	3.21 (0.88)	3.48 (0.76)	3.78 (0.88)	6.90**	SAD < HC***	0.65 [0.31, 1.08]
Forgetting	3.17 (0.91)	3.11 (0.83)	3.40 (0.93)	1.58; n.s.		
Problem solving	3.02 (1.07)	3.51 (0.83)	3.51 (1.16)	4.17*	SAD < HC*	0.44 [0.07, 0.83]
Reappraisal	2.74 (0.89)	2.63 (0.95)	3.06 (1.11)	2.66; n.s.		
Maladaptive						
Giving up	2.87 (1.03)	2.11 (0.73)	1.64 (0.60)	35.38***	SAD > HC***	1.46 [1.05, 1.93]
					SAD > SP***	0.82 [0.48, 1.25]
					SP > HC*	0.72 [0.33, 1.17]
Aggressive action	1.54 (0.71)	1.58 (0.63)	1.48 (0.56)	0.39; n.s.		
Withdrawal	2.79 (1.07)	1.76 (0.65)	1.62 (0.77)	31.84***	SAD > HC***	1.26 [0.88, 1.70]
					SAD > SP***	1.12 [0.75, 1.56]
Self-devaluation	3.11 (1.15)	2.29 (0.99)	2.69 (1.04)	7.21**	SAD > SP***	0.75 [0.31, 1.22]
Rumination	3.14 (0.98)	2.80 (0.97)	2.35 (0.89)	10.85***	SAD > HC***	0.85 [0.50, 1.24]
					SP > HC*	0.50 [0.06, 0.95]

*Notes.* *SAD* social anxiety disorder, *SP* specific phobia, *HC* healthy control

* *p* <.05

** *p* <.01

*** *p* <.001

### Size of ER Strategy Repertoire

Across all three groups, children and adolescents used between 6 and 7 ER strategies on average (*M* = 6.81, *SD* = 2.28). Table 4 shows descriptive information on the ER strategy repertoire for each group. At the first step of the hierarchical Poisson regression analysis (Table 5), none of the control variables were significantly associated with the ER strategy repertoire. At step two, neither of the two dummy-coded group variables were found to be significant predictors of the ER strategy repertoire. Given the non-significance of all predictors, the Pseudo $\:{R}^{2}$ values were also not significant, indicating that the model did not fit the data. Thus, after controlling for age, gender, and depressive symptoms, group membership was not a significant predictor of repertoire size.

**Table 4:** Descriptive analysis of overall ER strategy repertoire and group differences in ratios of adaptive and maladaptive ER strategies

	SAD (*n* = 60)	SP (*n* = 41)	HC (*n* = 63)			
	M (SD)	M (SD)	M (SD)			
ER strategy repertoire	6.53 (2.14)	6.80 (2.05)	7.08 (2.54)			
	SAD (*n* = 60)	SP (*n* = 41)	HC (*n* = 62 ^a^)	Statistics		
	*M* (*SD*)	*M* (*SD*)	*M* (*SD*)	*F*(2, 160)	Post hoc Tukey	*d* [95% CI]
Ratios in %						
Adaptive	50.09 (22.50)	65.27 (14.18)	71.95 (14.69)	23.63***	SAD < HC***	1.15 [0.77, 1.61]
					SAD < SP***	0.78 [0.43, 1.15]
Maladaptive	28.61 (20.25)	10.21 (11.60)	8.36 (9.89)	32.69***	SAD > HC***	1.28 [0.90, 1.72]
					SAD > SP***	1.06 [0.72, 1.47]
Other	21.30 (15.21)	24.53 (11.30)	19.69 (15.02)	–	–	–

*Notes.* *SAD *social anxiety disorder, *SP* specific phobia, *HC* healthy control

^a^ HC sample size after exclusion of one HC participant with ER strategy repertoire = 0

*** *p* <.001

**Table 5:** Poisson regression of ER strategy repertoire

Variables	Estimate	SE	95% CI	Pseudo$\:{R}^{2}$
Step 1: Control variables				0.03
Age	0.03	0.02	[–0.00, 0.07]	
Gender (female)	0.02	0.06	[–0.10, 0.14]	
CDI	–0.003	0.004	[–0.011, 0.004]	
Step 2: Group ^a^				0.03
SAD	–0.08	0.09	[–0.25, 0.09]	
SP	–0.03	0.08	[–0.18, 0.12]	

Values rounded to two decimals, except for coefficients smaller than ± 0.01, which are shown to three decimals. *CDI* Children’s Depression Inventory, *SAD* social anxiety disorder, *SP* specific phobia

^a^ dummy-coded with the HC group serving as the reference category

### Composition of ER Strategy Repertoire

**Fig. 1:**
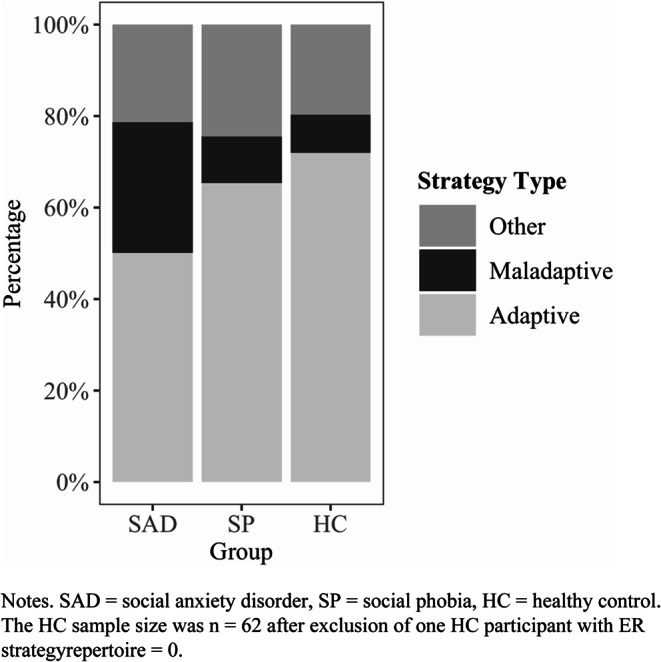
Composition of ER strategy repertoire for children and adolescents with SAD, SP, and HCs

Figure 1 shows the composition of the ER strategy repertoire from adaptive, maladaptive, and other strategies for each of the three groups. Results of the ANOVAs are presented in Table 4. The first ANOVA regarding the ratio of adaptive strategies in the overall repertoire revealed a significant main effect of Group, *F*(2, 160) = 23.63, *p* <.001, $\:{{\upeta\:}}^{2}$ = 0.23, indicating a large effect. Post hoc Tukey tests revealed a lower percentage of adaptive strategies for the SAD group compared to both the SP and the HC groups. The SP group and the HC group did not differ significantly in the percentage of adaptive strategies in the ER strategy repertoire.

Regarding the ratio of maladaptive strategies in the overall repertoire, the second ANOVA also revealed a significant main effect of Group, *F*(2, 160) = 32.69, *p* <.001, $\:{{\upeta\:}}^{2}$ = 0.29, which reflects a large effect. Post hoc Tukey tests revealed a higher percentage of maladaptive strategies for youth with SAD compared to both the SP and the HC groups. No significant difference was found between those with SP and HCs.

### Exploratory Analyses

Table 6 shows correlations between psychometric measures and sum scores of the adaptive and maladaptive subscales of the FEEL-KJ.

**Table 6:** Correlations between psychometric measures and sum scores of the adaptive and maladaptive subscales of the FEEL-KJ

	Adaptive ER strategy use ^a^	Maladaptive ER strategy use ^b^
Social anxiety ^c^	–0.27***	0.46***
Phobic fears ^d^	–0.19***	0.35***
Depressive symptoms ^e^	–0.35**	0.52***
General psychopathology ^f^	–0.23***	0.23***
Internalizing symptoms	–0.26***	0.30***
Externalizing symptoms	–0.17**	0.14*

*Notes.* Due to non-normality and ranks, Kendall’s τ was computed. Note that the German version of the questionnaires were used

^a^ sum score of the seven adaptive ER strategy subscales of the “Fragebogen zur Erhebung der Emotionsregulation bei Kindern und Jugendlichen” (FEEL-KJ)

^b^ sum score of the five maladaptive ER strategy subscales of the FEEL-KJ

^c^ revised Social Anxiety Scale for Children (SASC-R-D)

^d^ revised Fear Survey Schedule for Children (FSSC-R)

^e^ Children’s Depression Inventory (CDI)

^f^ Child Behavior Checklist (CBCL)

* *p* <.05

** *p* <.01.

*** *p* <.001

Across the entire sample, greater symptom severity in social anxiety, phobic fears, depressive symptoms, general psychopathology, internalizing, and externalizing symptoms were associated with lower adaptive and higher maladaptive ER strategy use. Effect sizes were low for general psychopathology and externalizing symptoms, low to moderate for social anxiety, phobic fears, and internalizing symptoms, and moderate to high for depressive symptoms.

We then computed rank-based partial correlations using Kendall’s $\:{\uptau\:}$ to examine the relationship between ER strategy use and social anxiety while controlling for depressive symptoms. The partial correlation between adaptive ER strategy use and social anxiety was non-significant, $\:{\uptau\:}$ = –0.075, *p* =.15, whereas the partial correlation between maladaptive ER strategy use and social anxiety remained significant, $\:{\uptau\:}$ = –0.228, *p* <.001.

## Discussion

The current study aimed to investigate the role of emotion regulation (ER) in youth with SAD, focusing on individual ER strategies but also the overall strategy repertoire, by comparing them to youth with SP and HCs. The inclusion of a homogenous clinical control group with SP allowed us to differentiate between shared and disorder-specific aspects of emotion dysregulation in anxiety disorders in children and adolescents. Moreover, we focused not only on the use of individual ER strategies, but also on the overall strategy repertoire and the ratio of adaptive and maladaptive ER strategies within the repertoire, as these aspects have so far received little attention in research on children and adolescents. As hypothesized, children and adolescents with SAD reported using adaptive ER strategies less frequently than both other groups. Contrary to our expectations, no differences were identified between the SP and HC groups regarding adaptive ER strategy use. All expected group differences were confirmed for maladaptive ER strategy use, with youth with SAD reporting the highest and HCs reporting the lowest use. Those with SP were found to be located between the other groups.

Although a broad ER strategy repertoire was hypothesized to be beneficial regardless of the adaptiveness of specific strategies, the groups unexpectedly did not differ in repertoire size. Finally, when combining the first two approaches by looking at the composition of the ER strategy repertoire, our hypotheses were partially confirmed. As expected and consistent with the results on the use of individual ER strategies, children and adolescents with SAD showed a lower ratio of adaptive and a higher ratio of maladaptive strategies in their ER strategy repertoire than both other groups. The SP and HC groups did not differ significantly regarding repertoire composition.

### Deficits in ER Strategy use in Children and Adolescents with SAD

Our study replicates previous research findings showing that children and adolescents with SAD use maladaptive ER strategies more frequently (Asbrand et al., 2016; Keil et al., 2017; Lange & Tröster, 2014) and adaptive ER strategies less frequently (Keil et al., 2017) than HCs. Consistent with these previous findings, a higher use of *rumination*, *giving up*, and *withdrawal* was found for maladaptive ER strategies. In addition to deficits in adaptive ER strategy use already reported by Keil et al. (2017), we also found a reduced use of more active, problem-oriented strategies like *problem-oriented action* and *problem solving* compared to HCs.

Overall, the picture emerges that youth with SAD have deficits in actively coping with their anxiety and its causes due to their withdrawing and resigning behavior. At the same time, the heightened negative repetitive thinking might also make it difficult for this group to accept anxiety and fearful events or to distract themselves and move on (Schmitz et al., 2010, 2011).

### Differences in ER Strategy use in Children and Adolescents with SAD and SP

Applying the RDoC perspective (Fernandez et al., 2016), we hypothesized that emotion dysregulation is a shared factor in several anxiety disorders, with the extent of ER deficits increasing from more circumscribed fears to more generalized conditions (Lang et al., 2016). We found a higher maladaptive and lower adaptive ER strategy use in youth with SAD compared to those with SP. Therefore, emotion dysregulation appears to be more pronounced in SAD as the anxiety disorder with more generalized symptoms and with often more extensive psychosocial impairment compared to SP. Youth with SP differed from HCs only regarding the use of maladaptive but not adaptive ER strategies, indicating a lower extent of ER deficits.

A possible explanation might be that a heightened use of maladaptive ER can already be detected at lower levels of psychopathology, whereas deficits in adaptive ER become apparent only in more severe and generalized disorders like SAD. This view is consistent with findings comparing youth with diagnosed SAD and those with heightened social anxiety without a diagnosis (Lange & Tröster, 2014). While they show similar deficits in maladaptive ER strategy use, only the group with diagnosed SAD significantly differed from HCs regarding adaptive ER strategy use. A meta-analysis by Aldao et al. (2010) also seems fitting in this context, as it found that maladaptive ER strategies were found to show a stronger association with psychopathology than adaptive ones. One of the explanations proposed for this finding takes into account the interaction between adaptive and maladaptive ER strategies, therefore considering the total ER strategy repertoire (Aldao & Nolen-Hoeksema, 2012b). We will return to this aspect when discussing repertoire composition, but will first address possible reasons for not having found group differences in the size of the ER strategy repertoire.

### No Difference in the Size of ER Strategy Repertoire

The fact that the groups did not differ in the size of their ER repertoires (number of used ER strategies) might imply that ER repertoire size may not be an indicator of emotion dysregulation that is relevant to SAD and other anxiety disorders in youth. Previous findings have already indicated that the sheer number of strategies in an individual’s ER strategy repertoire might not be the most relevant factor for well-being (Daniel et al., 2023; Goodman et al., 2021; Grommisch et al., 2020). Instead, it might be more important how well youth with SAD choose ER strategies that fit a given context or how skillful they are at implementing those strategies.

Another aspect that may have compensated for the expected group differences is that, compared to HCs and youth with SP, children and adolescents with SAD might experience more anxiety-provoking situations in their everyday lives, leading to more frequent attempts at ER with a variety of different strategies (Daniel et al., 2023). To further investigate this possible confounding aspect, strategy use and repertoire size of children and adolescents with SAD should be investigated in relation to the regulation of emotions other than anxiety.

Moreover, our operationalization of the repertoire through the FEEL-KJ limited the types of strategies assessed, making it difficult to compare the results to findings based on other methods of assessing ER strategy repertoire, like an interview about an anxiety-provoking event (Quiñones-Camacho & Davis, 2019). Furthermore, since self-report methods rely on the recall of past experiences and regulatory attempts, participants’ reports of their habitual ER strategy use might not truly reflect their everyday strategy use (Grommisch et al., 2020). Lastly, our results regarding the repertoire composition provide another plausible reason for the lack of group differences in repertoire size.

### Different Composition of ER Strategy Repertoire in Children and Adolescents with SAD

In line with our hypotheses, children and adolescents with SAD were found to have a higher ratio of maladaptive and a lower ratio of adaptive ER strategies in their repertoire compared to children and adolescents with SP and HCs. Consequently, the total number of ER strategies in the repertoire may have been balanced out and therefore did not differ significantly between the groups. The finding of a different repertoire composition is also consistent with our results regarding the use of individual strategies, which indicate a decreased frequency of adaptive and increased frequency of maladaptive strategy use in youth with SAD. Those with SP reported applying some maladaptive ER strategies more frequently than HCs, but the low overall use meant that these differences were not reflected in a different ratio of maladaptive ER strategies in their repertoire.

In considering how adaptive and maladaptive strategies interact with each other in the repertoire and how this might affect associations with psychopathology (Aldao & Nolen-Hoeksema, 2012b), two opposing hypotheses have been proposed. First, the interference hypothesis states that the use of adaptive ER strategies might be impaired for individuals who also exhibit a higher use of maladaptive ER strategies. In contrast, the compensatory hypothesis proposes that adaptive ER strategies may be most impactful and have the strongest negative associations with psychopathology in individuals who tend to frequently use maladaptive ER strategies (Aldao & Nolen-Hoeksema, 2012b). Our findings on repertoire composition do not allow for any definite conclusion on the validity of one of the two hypotheses; however, they might serve as a starting point for future studies. Given that the ER strategy repertoire of youth with SAD is characterized by a lower ratio of adaptive ER strategies, they may be lacking the compensatory effect of adaptive ER strategies that makes the maladaptive ones have less detrimental consequences. Thus, it could be insightful to compare the SAD, SP, and HC groups in terms of how predictive their adaptive strategy use is for symptoms of psychopathology, particularly anxiety. If the adaptive ER strategies show the strongest negative associations with symptoms in youth with SAD, being the group with the highest maladaptive ER strategy use, this could be taken as evidence for the compensatory hypothesis (Aldao & Nolen-Hoeksema, 2012b).

According to the interference hypothesis, however, the high levels of maladaptive strategy use in those with SAD might impair access to adaptive ER strategies and their implementation (Aldao & Nolen-Hoeksema, 2012b). Were this to be studied, the interference hypothesis would be supported if adaptive ER strategies in the SAD group had smaller or no negative associations with anxiety symptoms compared to the other groups.

Previous studies are scarce, especially in the context of SAD, but they tend to support the compensatory hypothesis (Aldao et al., 2010; McMahon & Naragon-Gainey, 2018). Together with our results showing a higher ratio of maladaptive ER strategies in the repertoire of youth with SAD compared to HCs and youth with SP, this may have important implications for the persistence of SAD: Drawing from a broad range of maladaptive ER strategies likely impairs effective ER. As these detrimental effects of maladaptive ER strategies cannot be compensated for by enough adaptive strategies, this might result in even more severe emotion dysregulation. Anticipating these difficulties, youth with SAD might increasingly avoid anxiety-provoking situations, thereby maintaining the disorder. Further studies with children and adolescents with SAD on the interaction of ER strategies in the repertoire are necessary, as findings provide important insights for SAD treatment.

### Clinical Implications

Although it has been proposed that specific ER components might increase treatment efficacy (e.g., Hannesdottir & Ollendick, 2007), an advantage of cognitive behavioral therapy (CBT) with a specific focus on emotion and ER over classic CBT in the treatment of anxiety disorders in youth has not yet been empirically proven (Suveg et al., 2018). Our results suggest that emotion-focused components should be tailored more to disorder-specific differences in emotion dysregulation, as ER deficits in SAD and SP varied in their extent. In addition to focusing on individual ER strategies, shifting the ratios in the repertoire by reducing maladaptive and increasing adaptive ER strategies could also be a helpful approach, especially in SAD treatment. This may not require eliminating the use of maladaptive ER strategies. Instead, future studies should examine whether increasing the ratio of adaptive ER strategies might compensate to a certain extent for the deleterious effects of maladaptive ER strategies (McMahon & Naragon-Gainey, 2018).

### Limitations and Future Directions

Some study limitations offer directions for future research. First, our cross-sectional design limits causal interpretations regarding the role of ER in the development of SAD, which should be investigated in further studies using a longitudinal design. It should also be noted that neither our study hypotheses nor the analyses were pre-registered. Furthermore, the FEEL-KJ does not provide information on the type of anxiety-provoking situations participants were recalling as well as the content of specific strategies (e.g., content of ruminative thinking), both of which likely differed between SAD and SP. These limitations could be addressed by examining ER strategies through an interview (Quiñones-Camacho & Davis, 2019). Internal consistencies of some FEEL-KJ subscales were low, which could be explained by the small number of items per ER strategy but may affect the validity of our conclusions. This is particularly relevant for the repertoire variable, where combining two weakly correlated items into a dichotomous score may limit construct validity. As described in the methods section, this operationalization approach was developed specifically for the present study and requires future validation. Relatedly, we did not include ER strategies in the repertoire variable that participants reported using rarely or even less frequently, but it could be argued that they are still accessible to children and adolescents. However, as the rating scale of the FEEL-KJ does not provide an option indicating that participants did not use a strategy, we had to establish a criterion based on frequency. It was our consensus based on conceptual considerations to only define ER strategies as part of the repertoire that participants applied at least occasionally in anxiety-provoking situations. The aforementioned interview method would address this limitation by making it possible to combine all mentioned ER strategies into a measure of the ER strategy repertoire. In general, it should be noted that studies have used a variety of approaches in the operationalization of the ER strategy repertoire, from single-quantitative indexes as in our case to person-centered approaches with profiles of usage patterns (e.g., Lougheed & Hollenstein, 2012). Furthermore, to gain a broader understanding of ER flexibility beyond the aspect of the repertoire size, ecological momentary assessment methods might be a useful approach to assess the ability to implement context-appropriate ER strategies. Another limitation concerns the fact that, in the current sample, higher symptom severity regarding fear-related measures, depressive symptoms, general psychopathology, internalizing, and externalizing symptoms, was associated with lower adaptive and higher maladaptive ER strategy use. The findings may reflect a broader influence of overall symptom severity, particularly depressive symptoms, on ER difficulties, rather than effects driven solely by anxiety severity. However, our analyses also indicate that especially maladaptive ER strategy use shows specific associations with social anxiety. Furthermore, while youth with SP reported higher levels of phobic fears and overall psychopathology compared to HCs, both control groups did not differ in their strategy repertoire composition. To clarify the role of symptom severity, future studies should include a clinical control group that closely matches the SAD group in terms of symptom severity and comorbidities (e.g., youth with generalized anxiety disorder).

Overall, our findings indicate that emotion dysregulation increases from circumscribed, fear-based to more generalized and highly comorbid conditions, as SAD is characterized by more pronounced ER deficits than SP, which is also reflected in a different repertoire composition. Further research on the interaction of adaptive and maladaptive ER strategies may help to determine whether a shift in the repertoire composition in favor of adaptive ER strategies could be a helpful new treatment approach. In any case, we encourage future studies on ER deficits in SAD to expand the view from individual ER strategies to the entire repertoire.
